# The Role and Challenges of Exome Sequencing in Studies of Human Diseases

**DOI:** 10.3389/fgene.2013.00160

**Published:** 2013-08-26

**Authors:** Zuoheng Wang, Xiangtao Liu, Bao-Zhu Yang, Joel Gelernter

**Affiliations:** ^1^Department of Biostatistics, Yale School of Public Health, Yale UniversityNew Haven, CT, USA; ^2^Division of Human Genetics, Department of Psychiatry, Yale University School of MedicineNew Haven, CT, USA; ^3^Comprehensive Cancer Center, The Ohio State UniversityColumbus, OH, USA; ^4^Department of Statistics, The Ohio State UniversityColumbus, OH, USA; ^5^Department of Genetics, Yale University School of MedicineNew Haven, CT, USA; ^6^Department of Neurobiology, Yale University School of MedicineNew Haven, CT, USA

**Keywords:** exome sequencing, exome arrays, Mendelian diseases, complex traits, whole-genome sequencing

## Abstract

Recent advances in next-generation sequencing technologies have transformed the genetics study of human diseases; this is an era of unprecedented productivity. Exome sequencing, the targeted sequencing of the protein-coding portion of the human genome, has been shown to be a powerful and cost-effective method for detection of disease variants underlying Mendelian disorders. Increasing effort has been made in the interest of the identification of rare variants associated with complex traits in sequencing studies. Here we provided an overview of the application fields for exome sequencing in human diseases. We describe a general framework of computation and bioinformatics for handling sequencing data. We then demonstrate data quality and agreement between exome sequencing and exome microarray (chip) genotypes using data collected on the same set of subjects in a genetic study of panic disorder. Our results show that, in sequencing data, the data quality was generally higher for variants within the exonic target regions, compared to that outside the target regions, due to the target enrichment. We also compared genotype concordance for variant calls obtained by exome sequencing vs. exome genotyping microarrays. The overall consistency rate was >99.83% and the heterozygous consistency rate was >97.55%. The two platforms share a large amount of agreement over low frequency variants in the exonic regions, while exome sequencing provides much more information on variants not included on exome genotyping microarrays. The results demonstrate that exome sequencing data are of high quality and can be used to investigate the role of rare coding variants in human diseases.

## INTRODUCTION

Determining the genetic basis of human diseases is one of the major research areas in medical science (McCarthy et al., 2008). The allelic spectrum of variants underlying human disorders has long been a topic of discussion and speculation (Pritchard, 2001; Reich and Lander, 2001). Despite significant progress in the identification of large numbers of loci that contribute to complex traits in genome-wide association studies (GWAS), only a small fraction of the observed heritability is explained by the confirmed (genomewide-significant) common variants (Manolio et al., 2009; Schork et al., 2009). A recent study (Yang et al., 2010) demonstrated that the heritability estimation can be improved by using all genomewide common single nucleotide polymorphisms (SNPs) relative to that using only identified genomewide-significant SNPs, and this accounts for some of the heritability that is “missing.” The advent of massively parallel sequencing technologies has transformed the field of human genetics and substantially reduced the cost of sequencing large genomic regions relative to the traditional Sanger sequencing (Mardis, 2008; Ansorge, 2009; Metzker, 2010). This allows researchers to investigate variants from a wide range of allelic spectrum, including variants that are too rare for inclusion on microarrays and new mutations; and higher-level structural variants. Thus, sequencing approaches have the potential to explain some of the missing heritability from GWAS for complex traits, through identification of rare variants and structural variations (Manolio et al., 2009; Eichler et al., 2010). However, it is still financially impractical, for most laboratories, to perform whole-genome sequencing for large numbers of subjects at sufficiently high coverage, in order to complete valid large-scale genetic association studies of complex traits.

A more economical approach to gene discovery is to focus on functional coding regions of the human genome. The exome represents about 1% of the human genome with approximately 30 million base pairs, but accounts for about 85% of mutations identified in Mendelian diseases (Ng et al., 2009). Recent developments in high-throughput sequence capture methods have made exome sequencing an attractive and practical approach for investigation of coding variation (Biesecker, 2010; Kaiser, 2010; Mamanova et al., 2010; Ng et al., 2010a, b). During the past 3 years, more than 100 genes have been characterized in rare Mendelian diseases by the use of whole exome sequencing. Application of this approach for non-Mendelian phenotypes has been, to date, much less widespread.

Traditional microarray-based tag SNP genotyping techniques designed for GWAS target relatively common variants. With the rich information gathered from sequencing over 12,000 individual exomes and whole-genome sequences representing multiple ethnicities and complex traits, the companies that market genotyping arrays (chips), Illumina, and Affymetrix, through a collaboration with leading geneticists, have designed exome chips that contain putative functional exonic variants, with the majority of them focusing on rare markers selected from sequencing studies (Exome chip design^1^). The introduction of exome arrays has provided a fast and economical platform for genotyping the included exonic variants, and has to some extent bridged the gap between traditional genotyping arrays and exome sequencing of very large numbers of samples, although they bring with them their own particular technical issues, most particularly, the inability to query very rare variants or new mutations.

Both exome sequencing and exome genotyping arrays are designed to investigate coding variation. The current approach for exome sequencing is based on a probe hybridization method to select the entire set of human exons as the sequencing target (Hodges et al., 2007; Gnirke et al., 2009). Although the exonic regions are the primary target, the efficiency of different capture technologies can affect the amount of information outside target regions. Currently, there is still a portion of captured DNA fragments falling into non-coding regions such as introns, intron-exon boundary regions, and intergenic regions – some of these regions often contain functional elements. A recent report (Guo et al., 2012) demonstrated that the small amount of sequencing data that lies outside the exonic target regions is of high quality and can be used in genetic studies. In contrast, exome arrays focus on a fixed set of variants by design. Therefore, exome sequencing, compared to the use of exome arrays, generates not only more genetic variations at base-pair resolution in the coding regions, but also additional, albeit limited, variant information outside the primary target regions. In this paper, we first provide an overview of the main application fields for exome sequencing relative to exome genotyping arrays in human diseases. Next, we describe the computational and statistical challenges for handling sequencing data. Then we evaluate the data quality and agreement between these two platforms using our exome sequencing and exome microarray data collected on the same set of subjects. Finally, we discuss some limitations of exome sequencing.

## APPLICATIONS OF EXOME SEQUENCING

Next-generation sequencing (NGS) technologies have been applied to several important areas including genomes, transcriptomes, epigenomes, and metagenomes (Zhou et al., 2010). Here, we mainly consider applications of sequencing to the identification of genes and mutations that influence risk for human diseases.

### MENDELIAN DISORDERS

The “traditional” approach to elucidating causes of Mendelian disorders – or in any event, the first generalizable approach to locate risk genes without prior knowledge – is based on linkage analysis followed by positional cloning (Botstein and Risch, 2003). Linkage studies require ascertainment of a sufficient number of probands with their families, and thus are not suitable for rare Mendelian diseases where only one or a few individuals may be sampled. In addition, modest-sized linkage studies are not sensitive enough to detect co-segregation within families in case of locus heterogeneity and phenotypic heterogeneity. NGS methods, on the other hand, have the potential to identify all kinds of genetic variation at base-pair resolution throughout the human genome in a single experiment (Bamshad et al., 2011; Gilissen et al., 2011; Ku et al., 2011), and provide an unbiased approach to detecting genetic variation within an individual. Currently sequencing instruments are still limited by throughput and cost efficiency. Exome sequencing, by capturing the protein-coding portion of the genome, generates a full picture of variation at functionally important regions of the genome (excluding regulatory changes), and has now become technically feasible and a more cost-effective strategy to work out the genetic basis of Mendelian disease. It has been a proven tool for the identification of *de novo* mutations underlying some rare monogenic diseases such as Kabuki syndrome (Ng et al., 2010a) and Miller syndrome (Ng et al., 2010b). Since November 2009, exome sequencing has led to the discovery of more than 100 genes in Mendelian diseases (Rabbani et al., 2012). As the sequencing cost per base will drop in the near future, we expect that whole-genome sequencing will be the ultimate approach to detection of *all* genomic variations and help us gain more knowledge on the genetics of Mendelian diseases – but even when the laboratory costs of generating full sequences decrease, there will still be very substantial informatics costs, which are also much lower of exome analysis.

### COMPLEX DISEASES

Over the past 8 years, the genetics research community has put a great deal of effort on studies of complex diseases which are caused by the interplay among multiple behavioral, environmental, and genetic factors. Association studies have been applied for decades to investigate the genetics of complex traits (Marian, 2012). With the advancement of high-throughput genotyping technologies, GWAS has been the main tool to find susceptibility genes based on the principle of linkage disequilibrium at the population level (Visscher et al., 2012). The development of SNP arrays genotyping hundreds of thousands or even millions of markers in a single assay has made GWAS feasible in large-scale population genetic studies. Since 2005, more than 8,000 loci have been reported to be associated with various human complex diseases and traits (A catalog of published GWAS^2^). The selection of markers investigated in most GWAS is based on the “common disease, common variant” hypothesis. SNP arrays provide a picture of genome-wide polymorphism in many individuals (The International HapMap Consortium, 2005, 2007), however, they inevitably suffer from ascertainment biases favoring SNPs that are common in the populations for variant discovery (Akey et al., 2003; Clark et al., 2005). In contrast, gene sequencing provides a more accurate and complete perspective with respect to all polymorphisms in target regions, or whole-genome (Tennessen et al., 2011). As a result, the field is now shifting toward the study of low frequency variants under the hypothesis of “common disease, rare variant,” i.e., multiple rare variants with large effect size are in some cases the main determinants of complex disease genetic risk (Marian, 2012). Exome genotyping arrays, based on the knowledge attained from many NGS studies, were designed also to target at a carefully selected subset of rare coding variants. Currently, exome arrays have served as a fast and economical tool for the initial investigation of the role of rare exonic variants in complex diseases (Huyghe et al., 2013), although more comprehensive evaluation of low frequency variants, copy number variants (CNVs), and structural variation, is accomplished much more effectively by NGS.

## COMPUTATIONAL AND STATISTICAL CHALLENGE OF SEQUENCING DATA

Next-generation sequencing instruments sequence millions of short DNA fragments in parallel. Compared to gene chip analysis, the data generated by sequencing require more sophisticated bioinformatics and statistical tools. In the identification of variants in NGS studies, the raw data are pre-processed into nucleotide base calls called short reads, varying from dozens to hundreds of base pairs, in the form of a FASTQ file. To call variants from sequencing data, many alignment methods and variant callers have been developed and used to create complex pipelines. A typical pipeline contains an aligner and a variant caller. The aligner maps each of the short reads to positions on a reference genome. The resulting sequence alignment is stored in a sequence alignment/map (SAM) or binary alignment/map (BAM) file (Li et al., 2009a). The variant caller identifies variant sites where the aligned sequences deviate from the known sequences at the reference position. The list of positions is recorded in a variant call format (VCF) file (Danecek et al., 2011). Further steps involve filtering and annotation to reduce variant sites to a smaller set of genes (when the sequence studied is exomic) with possible function and activity. We will now discuss these steps in detail and review the statistical strategies for identifying causal variants in human diseases.

### ALIGNMENT

“Alignment” is the step of matching short nucleotide reads to a reference genome. There are various software programs, either commercially available or freely distributed, that can be used to perform sequence reads alignment; to name a few, Bowtie/Bowtie2 (Langmead et al., 2009; Langmead and Salzberg, 2012), BWA (Li and Durbin, 2009, 2010), MAQ (Li et al., 2008), Novoalign^3^, and SOAP (Li et al., 2009c). There are many others that are more computationally intensive and are less frequently used. The performance of different alignment methods has been extensively studied (Bao et al., 2011; Ruffalo et al., 2011; Pattnaik et al., 2012). They are based on either hash tables or the Burrows–Wheeler transform (BWT; Burrows and Wheeler, 1994). The former hashes short reads or the reference genome into memory, while the latter compresses data features by creating an index of the reference genome to allow fast access of potential alignment locations (Nielsen et al., 2011). In general, BWT-based methods are faster and more memory-efficient. For instance, the BWA approach, based on BWT, provides a good balance between speed, memory usage, and accuracy, and is currently one of the most commonly used methods for alignment in sequencing projects.

As the current NGS technologies use PCR-like amplification steps in the library preparation, multiple reads originating from the same template could be sequenced. Overrepresentation of certain alleles due to amplification bias introduced during library construction tends to interfere with variant calling. For this reason, it is common to remove PCR duplicates after alignment in exome or whole-genome sequencing studies.

### VARIANT CALLING

After alignment of short reads to the reference genome, the next step in the bioinformatics process is variant identification. Currently the sequencing error rate is estimated to be about 1%, which is at a similar scale of the frequency of rare variants or higher. For genotype calling, the presence of sequencing error poses a computational challenge for the identification of true variants. Early generations of genotype calling methods counted allele at each position and used simple cutoff values to determine when to call a SNP. More recent probabilistic methods, such as MAQ (Li et al., 2008) and SOAPsnp (Li et al., 2009b), use fixed prior values for modeling heterozygote probability as well as sequencing error, and make genotype calls based on posterior genotype probabilities. Currently, some widely used variant calling methods include SAMtools (Li et al., 2009a), the Genome Analysis ToolKit (GATK, McKenna et al., 2010), and Atlas2 (Challis et al., 2012). SAMtools builds upon a revised MAQ model to perform computation of genotype likelihood and SNP calling. GATK utilizes the MapReduce (Dean and Ghemawat, 2008) functional programming technique for variant calling, SNP filtering, and quality recalibration. Atlas2 employs a logistic regression model trained on validated whole-exome sequencing data and has better power to assess the quality of potential variants (Ji, 2012).

We conducted a comprehensive evaluation of the variant identification methods using the exome sequencing data described in the next section. Based on our comparisons, GATK in general provided the highest quality of variant identification (Liu et al., Unpublished data).

Insertion and deletion (Indel) mutations are another common form of polymorphism. It requires gapped alignment and pair-end sequence inference. Several software packages have been developed to identify indels, including Pindel, a pattern growth method; and Dindel, a Bayesian approach. A detailed review on Indel calling has been published by Neuman et al. (2012).

There are several issues that can complicate the variant calling step. First, the presence of indels is a major source of false positive in variant identification. Alignment algorithms that allow for gapped alignments are preferred. Second, variable GC content in short reads, error introduced by library preparation due to PCR artifacts, and variable base quality scores can affect variant calling. The original quality scores assigned by the sequencer machine have been shown to be inaccurate and biased. Thus several SNP calling algorithms, like GATK and SOAPsnp, have recommended recalibration of base quality scores, using various calibrated error models to empirically estimate error rates for each base, in order to improve variant call accuracy.

### ANALYZING VARIANTS IN SEQUENCING

The main challenge of analyzing sequencing variants in human diseases is to identify disease-related alleles (which may be new mutations) accounting for a large number of non-pathogenic polymorphisms in the genome (Bamshad et al., 2011). Strategies for finding causal variants differ between Mendelian and complex diseases. Currently, successes in serious Mendelian disorders through exome sequencing rely on various heuristic filtering methods to reduce the number of candidate genes. First, the complete penetrance of a trait is usually assumed, i.e., all carriers of a disease-causing variant will have the phenotype. Any variants present in public databases such as HapMap (The International HapMap Consortium, 2005, 2007), 1000 Genomes Project (Abecasis et al., 2010), and dbSNP (Sherry et al., 2001) will be excluded from further consideration. Then on the basis of the mode of inheritance, for example, a recessive model, the list of candidate variants can be further reduced. This has successfully led to the identification of rare causal variants in more than 10 studies of recessive disorders. However, this type of filtering has certain limitations. Restricting the candidate variants to those not in public databases in the first filtering step could result in exclusion of possible pathogenic variants in the database, an especially noteworthy problem for the mapping of recessive traits. In addition, filtering based on complete penetrance can eliminate variants that are segregating in the population at low frequencies. Therefore more sophisticated analytical and filtering procedures that take into account the minor allele frequency (MAF) of the risk variant hold great promise to finding causal genes in Mendelian disorders (Stitziel et al., 2011).

To identify likely causal variants in complex traits, association tests are commonly employed. Sequencing studies enable us to investigate rare variants association with a trait under the assumption that multiple rare variants constitute the driving force for the trait of interest. The association with rare variants poses new statistical challenges. Power to detect an association with an individual rare variant can be very low because only a small percentage of study subjects carry a rare variant. To increase statistical power, many groups have investigated aggregating sets of rare variants within a gene or genomic region to enrich association signals (Li and Leal, 2008; Madsen and Browning, 2009; Han and Pan, 2010; Morris and Zeggini, 2010; Price et al., 2010; Ionita-Laza et al., 2011; Lin and Tang, 2011; Wu et al., 2011), and recent studies show that power to detect rare variant effects can be greatly enhanced. A comprehensive review on the statistical methodology of sequence-based association studies is described by Ionita-Laza et al. (2013). Another important aspect in sequencing-based association studies is the choice of an appropriate study design. Population-based and family-based designs are the two most commonly used approaches in genetic association studies. For rare variants with large effect size, family-based designs can be advantageous because a particular rare variant found in an affected individual, if it is not a new mutation, is more common in that individual’s family than in subjects randomly sampled in the population; this design can therefore potentially enrich for genetic effects. Trio designs, and some other family designs, are also robust to population structure (Ott et al., 2011). However, it can be more difficult to ascertain samples for family-based designs compared to population-based designs. For different study designs, the analytical strategy for rare variant association needs to be chosen accordingly.

Above, we describe a general framework of computation and bioinformatics for handling sequencing data. Next we demonstrate data quality and agreement between exome sequencing and exome microarray (chip) genotypes using our data collected on the same set of subjects in a genetic study of panic disorder.

## DATA DESCRIPTION

We studied whole exome sequencing data on 20 patients of panic disorder collected at Connecticut VA Medical Center (VAMC). Twelve of these were from a single pedigree of five generations with more than 70 family members (not all of whom could be genotyped), and the rest were unrelated. All patients gave informed consent approved by the institutional review boards at Yale and CT VAMC. We studied all samples by exome capture using the NimbleGen SeqCap EZ exome v2.0 kit, which targets 44.1 Mb of the genome by design; samples were sequenced at the Yale Center for Genome Analysis (YCGA). DNA fragments from the 20 samples were barcoded and sequenced on five lanes of a flowcell (four samples per lane). The exome sequence data were 74-base paired-end reads generated from the Illumina HiSeq system.

Reads were aligned to the UCSC reference human genome assembly hg19 using the sequence alignment software BWA version 0.6.1 with the default parameters. The mapping files in SAM format were converted to the BAM format and sorted by SAMtools version 0.1.18. Local realignment around the known indels was performed by GATK version 1.6.9 on the sorted BAM files. Picard tools version 1.5.3 was used to remove PCR duplicates. Finally, base quality score recalibration was performed using GATK. These steps generated BAM files ready for variant calling. We used GATK for variant identification. Then the raw variants were filtered using VCFtools version 0.1.7. We further applied genotype filtering using depth ≥5 and genotype quality score ≥20 (Guo et al., 2012).

The 20 samples were also interrogated for 247,134 variants using the Illumina HumanExome Beadchip genotyping microarray. More than 90% of variants on the exome array fall in the human RefSeq exons. The majority of them are non-synonymous single nucleotide variations. The Illumina exome chip also contains a small fraction of SNPs in splice sites, selected synonymous SNPs, tag SNPs for previous GWAS hits in a variety of diseases, and ancestry informative markers (AIMs). Eight samples failed the genotyping quality control step were excluded from further analysis.

## RESULTS

**FIGURE 1:**
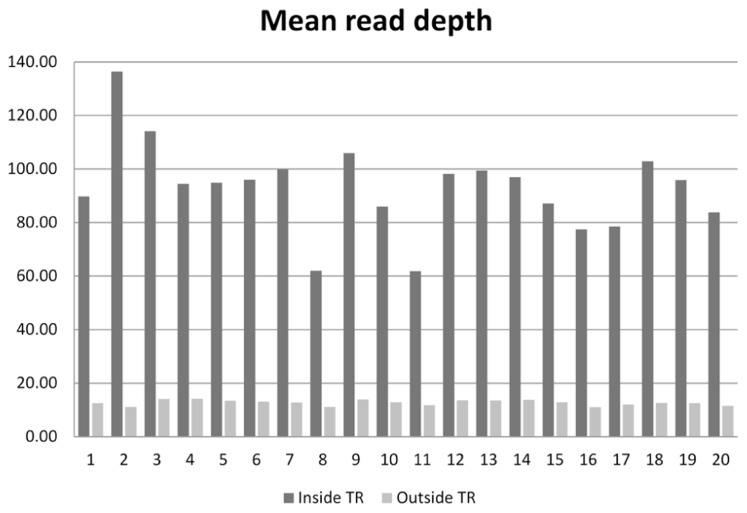
**Mean read depth inside the target regions and outside the target regions on the 20 sequenced subjects**.

On the 20 samples, we obtained an average of 48.7 (range 31.0–77.6) million reads per subject, with 93× mean depth in the target regions. The total length of the target region was 47.1 Mb, of which 34.1 Mb were exomic. On average, 95.9% (94.3–97.2%) of reads were mapped to the human reference genome. After removal of PCR duplicates, 90.7% (87.4–93.0%) of reads were retained. Among those uniquely mapped, 58.8% (55.7–62.9%) of reads were within the exonic regions. This proportion is similar to the numbers reported for Agilent’s SureSelect v1 and Illumina’s TrueSeq capture kits (Guo et al., 2012). The coverage for the target regions was as follows: 40.6 Mb (57.9%) had coverage of at least 1×, 33.9 Mb (48.4%) had coverage of at least 10×, and 32.4 Mb (46.3%) had coverage of at least 15×. For sequences outside the target region, 209.8 Mb were covered by at least 1 read, 40.5 Mb were covered by at least 10 reads, and 33.9 Mb were covered by at least 15 reads. The comparison of the average read depth inside and outside of the targeted exome is displayed in **Figure 1**. As we expected, the depth of coverage in the exome regions was higher in most regions due to target enrichment. An interesting feature regarding read depth is that it varied across subjects in the target regions, but stayed similar outside the target regions.

**FIGURE 2:**
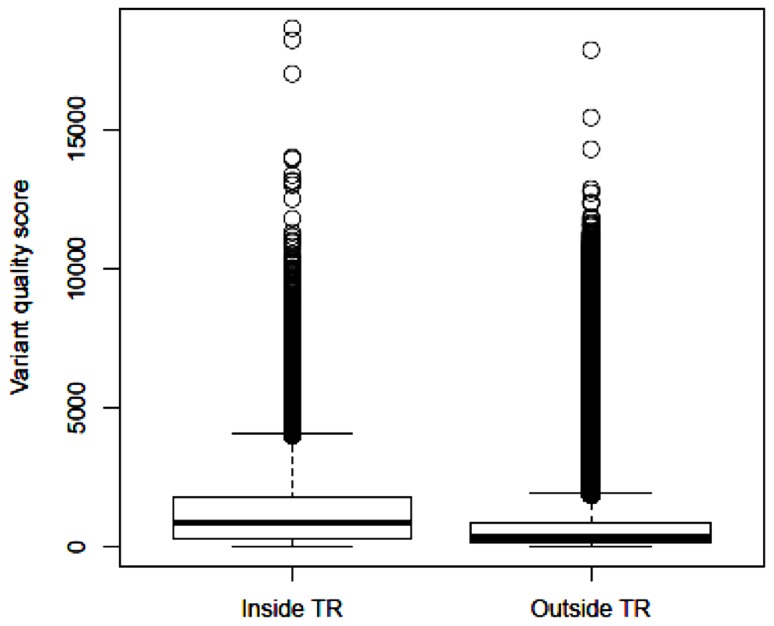
**Boxplot of identified variant quality scores inside the target regions and outside the target regions**.

After applying GATK and variant filtering, we identified an average of 26,082 (24,122–28,058) variants per subject inside the target regions, with Ti/Tv ratio of 2.85 (2.80–2.95). In addition, we observed an average of 63,760 (51,414–83,835) variants per subject outside the target regions, with Ti/Tv ratio of 2.17 (2.14–2.20). These results are close to the reports that the expected Ti/Tv ratio is around 3.0 for variants inside exons and about 2.0 elsewhere (Bainbridge et al., 2011). The median quality score of variants inside the target regions is 875.4, more than twice of the median quality score of 340.0 outside the target regions. Based on the distribution of variant quality scores inside and outside the target regions (**Figure 2**), the variants identified within the exome regions are of higher quality relative to those outside the target regions.

Besides variant quality score, another way to measure data quality for sequence-based variant calling is to investigate genotype concordance using an alternative genotyping platform. We use the exome microarray data for this purpose. Among the 12 subjects passed quality control on exome arrays, we identified 32,616 (13.2%) variant sites that showed at least one variation, i.e., at least one subject had a heterozygous genotype (denoted by 0/1) or homozygous rare allele genotype (denoted by 1/1). We compared concordance between the array genotypes and the sequence-based genotype calls. We calculated the genotype consistency rate between exome sequence-based and exome chip-based SNP calls for variants overlapping the two platforms in our samples. We used two types of consistency rate: overall consistency and heterozygous variant consistency. Heterozygous consistency rate was defined as the ratio between the number of heterozygous genotypes consistent between exome chip and exome sequencing and the number of heterozygous genotypes on the exome chip that had sequence-based calls with genotype quality score ≥20 and depth ≥5. The results for the 12 subjects are shown in **Table 1**. The overall consistency rate with array-based variant calls was >99.83% in all samples, and the heterozygous consistency rate was 98.14% (97.55–98.56%). The actual overall consistency rate is higher because we observed a large portion of concordant genotype calls between these two platforms falling in the category of homozygous reference genotypes. On average, more genotype calling errors would occur when the underlying genotype contains the allele that is not the reference allele. Depending on the purpose of the study, for example, in gene-trait association studies, the goal is usually to search for putative rare variants that could be causal for the trait; then, the heterozygous SNP calls would be more informative and the consistency measure based on heterozygous SNPs would be more representative of the true error rate. We also found that the consistency rate in the 1/1 genotype category was similar to the heterozygous consistency rate in our dataset.

**Table 1:** Results of genotype consistency between exome sequencing and exome chip on 12 subjects.

	Consistent genotypes			Consistency rate	
Subject	0/0	0/1	1/1	Overall (%)	Heterozygous (%)
1	218523	4686	2949	99.84	98.28
2	220203	4860	3092	99.83	98.56
4	218043	4608	3016	99.87	97.88
5	218478	4654	2976	99.84	98.21
6	218463	4685	2870	99.83	98.07
7	218553	4765	2999	99.85	97.96
8	214082	4625	2815	99.86	97.55
9	219050	4888	2892	99.87	98.33
15	218233	4719	2995	99.85	98.25
16	217804	4579	3024	99.83	97.99
17	217440	4654	2931	99.84	98.39
19	219067	4566	3025	99.83	98.26

Overall, the genotype calls generated by exome sequencing and exome genotyping arrays showed high agreement in all the 12 samples.

## DISCUSSION

We have provided an overview of the application of exome-focused NGS technologies in human diseases. The growing number of exome sequencing studies demonstrates the power of this approach in mapping genes involved in Mendelian disorders and suggests utility for complex traits as well. In many successful studies, a small number of individuals was analyzed, and often only affected individuals have been sequenced. However, there are still a large number of Mendelian diseases with unknown genetic causes.

Although exome sequencing has generated high-quality data for single nucleotide variant detection with sufficient depth of coverage, it is still difficult to detect accurately indels with short sequence reads generated by NGS technologies. In addition, exome sequencing is not suitable for the identification of structural variants and chromosomal rearrangements that may involve non-exonic sequence. Furthermore, as the current sequence capturing methods suffer from the problem of uneven and incomplete exonic region capture (Parla et al., 2011), potentially interesting mutations in these exonic regions could be missed. This will likely be solved in the future when the cost of whole-genome sequencing is lower.

Studies of genetically complex traits have also benefited from exome sequencing since the advent of NGS technologies. Although the small sample sizes that can be used in Mendelian diseases are underpowered for detecting association using currently available association tests for complex traits, we can still gain insight by studying small cohorts from the extreme ends of the phenotypic spectrum of common traits, and as costs come down, well powered studies of complex traits via exome sequencing have become feasible. This has been demonstrated by a successful example of a whole exome sequencing study of patients with extremely low levels of low-density lipoprotein (LDL) cholesterol (Musunuru et al., 2010). The findings of risk alleles in GWAS typically cannot pinpoint causal variants, but exome sequencing studies enable more accurate and complete variant discovery (of course this is under the assumption that the risk variant is exomic) and allow for, in theory, the direct association between phenotype and causal variant. They have provided a new mechanistic perspective on the development of the complex disease gene mapping paradigm. Currently, with sequencing data, there is still a strong demand for more powerful and efficient analytic methods for novel gene discovery in the analysis of complex diseases.

We demonstrated the high quality of exome sequencing data in our samples collected from a study of panic disorder. We examined SNP quality within and outside the targeted exome regions. With the NimbleGen SeqCap capturing method, about 59% of the reads in our dataset were mapped within the target regions, meaning and there are still a significant number of reads that map elsewhere. About 30% of reads fall outside >200 bp of the exonic region, and 10% of reads are within 200 bp from the nearest target region. Variant call qualities were generally better for positions within the target regions, due to successful target enrichment. Furthermore, we computed genotype concordance with exome microarray data. The overall consistency rate was >99.83% and the heterozygous consistency rate was 98.14%, which suggests that the two platforms maintained a large amount of agreement over low frequency variants in the exonic regions.

Undoubtedly, the data generated in NGS technologies will continue to grow in terms of the depth per individual and the number of samples per dollar. The role of computation and bioinformatics becomes more and more crucial in the analysis and interpretation of sequencing data. Tremendous effort has been devoted to the development of tools for variant analysis in the process of quality control, alignment, variant identification, and downstream association studies. As whole-genome sequencing becomes prevalent in the next few years, future developments of workflow and pipelines will facilitate researches on human diseases.

## Conflict of Interest Statement

The authors declare that the research was conducted in the absence of any commercial or financial relationships that could be construed as a potential conflict of interest.

## Footnotes

1

2

3
